# Healing of the Canoe: Preliminary Suicide Prevention Outcomes Among Participating and Non-Participating Youth

**DOI:** 10.1007/s11121-025-01806-x

**Published:** 2025-04-28

**Authors:** Tamara Perkins, Brian Lee, Juliette Mackin, Dennis Donovan, Stephanie Craig Rushing, Colbie Caughlan, Amanda Gchachu Kakuska, Leanza Walker

**Affiliations:** 1 NPC Research, Portland, OR USA; 2 Department of Psychiatry & Behavioral Sciences, University of Washington School of Medicine, Seattle, Washington USA; 3 Northwest Portland Area Indian Health Board (NPAIHB), Portland, OR USA; 4 Independent Contractor for NPAIHB, Portland, OR USA

**Keywords:** Indigenous, Native, Youth curriculum, Suicide prevention, Mental health, Resilience, Culture, Hope, Optimism, Youth surveys

## Abstract

Healing of the Canoe (HOC) is a community-derived, culturally grounded, and flexible curriculum for Native youth that builds a connection to culture and community and teaches skills that increase participants’ feelings of hope, optimism, and self-efficacy. This exploratory study is the first to examine self-reported survey outcomes after the addition of suicide prevention and intervention modules into the curriculum and includes a comparison group of similar youth. Repeated measures analysis of variance (rANOVA) models examined changes in scores from the Pre-Survey to the Post-Survey for an intervention group and a comparison group of youth in 2018–2019. Survey responses were combined into composite scores for the following categories of interest: hope, mental health, help-seeking and helping, suicide attempts, culture, and resilience. There were 74 youth in the intervention group (IG) and 59 in the non-intervention group (NIG) who completed both Pre- and Post-Surveys. The IG experienced significant positive effects on the hope and resilience scales from Pre- to Post-Survey, while the youth in the NIG saw a decrease in these same scales from Pre- to Post-Survey. The NIG experienced worse outcomes for mental health, while the IG saw a slight improvement. Despite a small number of youth participants and the naturalistic setting, which limited the ability to control for potential confounding variables, the results from this preliminary study are promising. Future studies with larger numbers of youth and more ability to account for additional factors may potentially show even more benefits of the HOC curriculum.

## Literature Review

Cultural strength or “collective resilience” is a potent resource deeply rooted in American Indian and Alaska Native (AIAN) communities that supports both collective and individual-level mental wellness (Teufel-Shone et al., 2018). Interventions that foster collective resilience naturally enhance individual protective factors through strengths-based teachings embedded in culture and traditional lifeways (Cwik et al., 2019). In fact, AIAN cultural connectedness is so crucial to the physical and mental well-being of Native peoples that it can be considered a social determinant of health (Masotti et al., 2020). Community-driven, culture-based approaches can help to counter the many risk factors underlying poor individual and community outcomes, including the continuing impact of colonialist and racist beliefs, policies, and practices on Native peoples in North America (Burnette et al., 2019; Wexler et al., 2024). Disparities in mental health experienced by many AIAN people today are just one consequence of such risk factors.

The urgent need to address AIAN youth well-being is evident in the statistics: AIAN youth are disproportionately represented among those having suicide risk, ideation, attempts, and death compared to other ethnic groups (Stone et al., 2022). According to the U.S. Centers for Disease Control and Prevention (CDC) data for 2018–2021, the suicide rate for non-Hispanic AIAN youth 10–24 is more than 3 times higher than that for all youth in this age group (CDC, May 2024). Additionally, during the 2009 to 2018 period, an estimated 14 to 27% of non-Hispanic AIAN adolescents attempted suicide (Mpofu et al., 2022).

In recent years, Native researchers, practitioners, and policy makers have been able to argue forcefully against “evidence-based” interventions premised on randomized control trials normed on non-Native peoples. Instead, they have advocated for utilizing millennia of cultural wisdom as the way to heal their communities from colonialist legacies and to thrive (Nebelkopf et al., 2011; O’Keefe, 2019). Culturally specific and Tribally contextualized interventions to address mental health generally—and suicide specifically—for AIAN youth suggest that local culture is a major protective factor in suicide prevention among AIAN individuals (Asher BlackDeer & Patterson Silver Wolf, 2020; Allen et al., 2022). Such approaches that incorporate Native “culture as intervention” promote sobriety and reasons for living in AIAN youth (Allen et al., 2018; Barlow et al., 2023).

Findings from several studies reinforce this perspective. Cwik et al. (2015) found that, along with a reduction in depression, participation in cultural activities was a major source of resilience among at-risk AIAN adolescents. Johns Hopkins researchers found that Tribal strengths and Indigenous knowledge and practices promoted health and physical, mental, emotional, and spiritual well-being in AIAN communities, with culture identified as a protective factor against youth suicide (O’Keefe et al., 2022). Doria et al. (2021) stressed the importance of incorporating traditional knowledge, increasing connection to both culture and community, and engaging with, and learning from, Tribal Elders as intervention components. AIAN youth benefit from such programs by allowing them to connect to cultural teachings (Hunter et al., 2022), which anchors them in their community, culture, and history.

## Healing of the Canoe

The Healing of the Canoe (HOC) curriculum is a 14-chapter community-derived and culturally grounded intervention specific to the Native canoe culture of the Pacific Northwest (PNW) in North America. The core HOC curriculum focuses on the following 10 concepts and life skills: self-awareness, recognizing and standing up against stereotypes, getting help from the community, mentoring others, coping with negative emotions, goal setting, overcoming obstacles and solving problems, listening/effective communication, understanding the consequences of substance use, and serving the community (Donovan et al., 2015). Trained Tribal prevention and youth-serving staff provide most of the core curriculum and invite Tribal culture keepers, community members, Elders, and/or Tribal Council members to teach a skill (like carving a dugout canoe), tell stories, or pair up with a youth in an activity meant to cultivate positive connections to community adults.

HOC was developed for Tribal youth through a collaboration between the Suquamish Tribe, the Port Gamble S’Klallam Tribe, and the University of Washington Alcohol and Drug Abuse Institute (ADAI), using community-based and Tribally-based participatory research methods. A key feature of the HOC curriculum is that it was designed to be flexible and adapted to each particular cultural, geographic, and social context in which it is delivered. More information about HOC can be found at https://healingofthecanoe.org/curriculum/.

The original HOC curriculum was focused on decreasing substance use and other risky behaviors. In the one published evaluation of HOC to date, the curriculum was found to result in increased hope, optimism, and self-efficacy across both a high school setting and intensive intervention workshops (Donovan et al., 2015). In 2016, the HOC curriculum was amended by its developers to add suicide prevention and intervention modules at the request of the Northwest Portland Area Indian Health Board for a grant project. One module focuses on helping youth understand their emotions and introduces more self-regulation skills that, along with all the cultural pieces, intend to increase participants’ feelings of hope, optimism, self-efficacy, and resilience. The suicide intervention module presents the warning signs of suicide in others and what to do if one recognizes that someone might be thinking of suicide using a “person overboard” canoe-based metaphor.

This study represents the first opportunity to evaluate the efficacy of HOC as a suicide prevention curriculum and includes a comparison group of similar youth. It is important to note that this evaluation was not planned as a rigorous study. Instead, the original intent of the HOC Pre- and Post-Surveys described here was to provide actionable evaluation information for program improvement to a convenience sample of Tribal communities that were then part of a grant program. At the time, there was no infrastructure, funding, or buy-in to set up a comparison group or aggregate the results to include multiple Tribal communities. However, the project team was able to identify and secure approval from two additional Tribal communities that implemented Pre- and Post-Surveys nearly identical to the HOC surveys. These communities had planned to provide the HOC intervention but did not.

While this study was conducted in a naturalistic setting with many uncontrolled variables, it still offers valuable insights as a natural experiment. This exploration is particularly important given the lack of research on HOC’s effectiveness following the addition of suicide prevention and intervention modules. Despite its limitations, the study provides a unique opportunity to examine real-world outcomes and potentially guide future, more controlled research in this critical area.

## Methodology

### Statistical Model

This repeated measures design examined the difference between measures observed at two distinct points in time for non-independent subjects. Repeated measures analysis of variance (rANOVA) was used to analyze the effect of the HOC intervention on each composite category score. While rANOVA is often cited in analyses involving observations over three or more points in time, it is used here over two points in time to ensure that the non-independence of events does not interfere with statistical validity. With two points, the sphericity assumption of rANOVA becomes trivial, and sphericity is assumed. While testing over only two means with rANOVA generally produces similar results to a paired *t*-test (Schober & Vetter, 2018), the use of rANOVA in this study allows for the control of the continuous age covariate. Further, the examination of the estimated marginal means produced by the rANOVA model allows for equivalent comparisons across gender, AIAN status, and Adverse Childhood Experiences (ACE) score factors. In this way, statistical power is preserved without the possibility of an increase in type II error due to incomplete cases (Muhammad, 2023).

All survey respondents in the study were part of one of two groups, the Healing of the Canoe (HOC) intervention group (IG) and the non-intervention group (NIG) [comparison group]. IG participants took a Pre-Survey immediately prior to the intervention and the same survey immediately after the intervention (Post-Survey) between March 2018 and August 2019 (depending on the timing of the intervention in their Tribe). The surveys were administered by trained Tribal and evaluation team staff. The NIG took the Pre- and Post-Surveys between February 2018 and June 2019. The responses were divided into the following categories: hope, resilience, mental health, help-seeking and helping, physical health, peer, school, family, community, and culture. A mean composite score was made for each category for the Pre-Survey and, similarly, for the Post-Survey. Additional variables were included in the rANOVA to allow for the examination of these measures while controlling for demographics such as age, gender, AIAN status, and ACE scores.

### Setting

All six Tribal communities in the sample are located in the PNW, have between 1000 and 3000 enrolled Tribal members currently living in the area, and have a long history of canoe culture as a cultural touchstone. HOC was delivered (or planned to be delivered) by Tribal staff primarily from Tribal education, Tribal youth services, and/or Tribal prevention departments in community centers, schools, on ancestral lands, and/or waterways. The initial IG HOC implementations were, as mentioned before, part of a multi-site grant project that funded both the implementations and basic evaluation (the Pre- and Post-Surveys). The four IG HOC implementations occurred in a variety of formats: school-based, a three-weekend session in the late spring and summer, a weeklong summer camp, and a month-long summer camp (the average length of HOC implementation was 38 days, *SD* = 48 days). Despite repeated efforts to document exactly how HOC was implemented in the four IG communities—for example, what order the modules were presented in, how long the presentation of each module was, youth attendance records, and any successes or challenges that came up in the implementation of each module—this information was not available.

The two NIG comparison Tribes were part of yet another (pilot) project in the PNW. In spring 2018, one of the two NIG Tribes planned to implement a newly adapted version of HOC that included their own specific Tribal stories and practices, and they identified a second Tribe to be a comparison group. Because the final HOC adaptation was not completed in time to pilot in spring 2018 as planned, the 2018–2019 set of Pre- and Post-Surveys was used as baseline data collection for the multi-year grant project (the average time from Pre- to Post-Survey was 14.8 months, *SD* = 1.6). Due to several delays (including long Tribal closures during COVID- 19), the adaptation was completed several years later. Although the adapted HOC curriculum was not implemented as originally planned, the baseline Pre- and Post-Surveys surveys for both Tribes have become the NIG for the purposes of this paper.

## Measures

The HOC Pre- and Post-Surveys are designed to measure the potential impact of the HOC curriculum. The surveys were developed collaboratively by the Tribal Health: Reaching out InVolves Everyone (THRIVE) program staff at the Northwest Portland Area Indian Health Board (NPAIHB) and NPC Research, with consultation from the ADAI and the Healing of the Canoe organization (https://healingofthecanoe.org/).

The Pre- and Post-Survey measures are grounded in the **Social Ecological Model (SEM**), which describes youth feelings and behaviors as having both individual and wider social context components and has been the corner-stone for recent AIAN suicide prevention research (Barlow et al., 2023). SEM has developed over at least 50 years, starting with Bronfenbrenner’s lifetime work on child and human development (1974, 1979, & 1994, to name just a few), then moving into prevention (e.g., Catalano et al., 2002), health and mental health promotion (e.g., Erickson et al., 2024; Krug et al., 2002; Stokols, 1996), and violence prevention (e.g., CDC, April 2024). This is the multi-level, strengths-based context in which the HOC curriculum is situated and necessitates collecting youth outcomes related to the various levels. For example, a youth’s feelings of isolation or joy are in a multifactor dynamic with the wider social context of peers, school, family, culture, and community.

The study team is composed of program-focused applied researchers helping Tribal programs understand the impact of the HOC curriculum with a Pre/Post-Survey. The SEM domains measure areas that are important for youth well-being. For example, Pre/Post-Surveys reveal whether the youth have Elders in the community that they can talk to, if they feel safe in their home, if they are getting enough sleep, etc. The results lead to celebrating successes as well as recommendations for Tribal site staff to consider in strengthening youth well-being, such as reviewing results with youth to discuss why they might not be getting enough sleep, what teachers and administrators do not know—but should know—about AIAN youth in their schools, etc.

The HOC surveys draw from valid and reliable measures, including the Children’s Hope Scale (Snyder et al., 1997), Youth Risk Behavior Survey (CDC, 2013), the Communities that Care Survey (Arthur et al., 2002), and the Voices of Indian Teens Survey (Moran et al., 1999). In 2017–2018, the Child and Youth Resilience Measure (Liebenberg et al., 2013) and a youth version of the ACE questions (National Council of Juvenile & Family Court Judges, 2006) were added to the HOC surveys by request from Tribal communities. The evaluation team occasionally made small adjustments in question or response option wording for clarity and accessibility.

The study team created four additional questions because existing measures in the literature could not be identified. These questions are about help-seeking, seeking help for others, and confidence in one’s ability to help someone who might be thinking of suicide. The team also added one open-ended question that asks about the most useful thing that youth learned in HOC.

The primary outcomes hypothesized to be common to the HOC intervention sites are increased protective factors at the individual level that prevent suicide: increased feelings of hope and optimism, increased sense of mental well-being, increased resilience, help-seeking, helping others get help, reduced suicide attempts, and increased feelings of connection to culture. Additionally, the effects of connections to physical health, peers, family, school, and community were explored since these might be impacted by the HOC curriculum (as well as being potential moderators of the individual-level measures). Thus, from the larger perspective of the SEM model and from the holistic and flexible nature of the HOC curriculum implementations (which could focus on any/all of the domains), the variety of measures across all the SEM domains are relevant—even if they did not produce notable findings here.

For each of the domains, two sets of scale scores were created for the Pre-Surveys and Post-Surveys collected from IG youth before and after delivery of the HOC curriculum in 2018 (two summer intensive interventions) and 2019 (one school-based and one summer intensive). The NIG youth Pre- and Post-Surveys were collected between February 2018 and June 2019. Each of the models controlled for age, gender, ACE score, and AIAN status (either self-identified as AIAN or not, regardless of the fact that they are members of the Tribal community). Primary outcome variables included the following:

### Hope

The responses from the six survey questions pertaining to the Children’s Hope Scale (Snyder et al., 1997) were averaged together to compile this composite score. Respondents were asked to rate six statements on the following scale: (1) none of the time; (2) a little of the time; (3) some of the time; (4) a lot of the time; (5) most of the time; and (6) all of the time. The six statements included “I think I am doing pretty well,” “When I have a problem, I can come up with lots of ways to solve it,” and “I think the things I have done in the past will help me in the future.”

### Mental health

A mental health score was based on the response to the question, “In general, how good is your mental health?” Youth were asked to rate their mental health on the following scale: (1) poor; (2) fair; (3) good; and (4) excellent. This question was adapted from the CDC Youth Risk and Behavior Survey (YRBS) question (CDC, 2013).

### Resilience

The Child and Youth Resilience Measure 12-Item scale (CYRM- 12) is the resilience scale used in this study (Liebenberg et al., 2013). The CYRM- 12 was developed and validated for use across diverse cultures and communities, including Indigenous communities, and now has been used with youth around the world. Respondents were asked to rate these statements on the following scale: (1) not at all; (2) a little; (3) somewhat; (4) quite a bit; and (5) a lot. Scores for each of the 12 items on the scale ranged from one to five, and from these, an average resilience score, also ranging from one to five, was calculated. Topics covered by the 12 statements included understanding youth self-efficacy, problem-solving, help-seeking, belonging, culture, etc.

### Help-seeking and helping

A composite help-seeking and helping score was calculated from three questions that were developed specifically for the HOC surveys by the study team because similar measures could not be found in the literature. Responses from three survey questions pertaining to suicide prevention were averaged together to compile this composite score. Respondents were asked to rate two statements on the following scale: (1) very unlikely; (2) somewhat unlikely; (3) somewhat likely; and (4) very likely. These statements included: “How likely would you be to seek help if you were feeling depressed or suicidal?” and “How likely would you be to seek help for a friend who you thought might be depressed or suicidal?” Respondents were also asked to rate the third statement, “Do you feel confident that you could help a friend or family member who is thinking about suicide?” on the following scale: (1) not confident at all; (2) not confident; (3) confident; and (4) very confident.

### Suicide attempts

Both IG and NIG surveys shared an item about past 12-month suicide attempts taken from the CDC YRBS. This question has been on the YRBS for over 20 years (CDC, 2023 with a discussion of validity and reliability in CDC, 2013). The IG Survey has additional items about past 30-day thoughts of suicide and past 30-day suicide attempts that were not included on the NIG version of the survey because suicide prevention was not the focus for those sites.

Additional measures in the SEM model—peers, school, family, community, culture, physical health, etc., are not reported here due to unremarkable outcomes (Table 1).

**Table 1:** Baseline (pre-survey) mean scores

Baseline scores	NIG		IG		*t*	df	*p*
	M	SD	M	SD			
Hope	4.34	0.90	4.12	1.02	1.31	128.04	.19
School	3.00	0.50	2.94	0.57	0.70	125.85	.49
Family	3.47	0.42	3.34	0.49	1.71	129.10	.09
Physical health	3.12	0.66	2.99	0.72	1.13	124.74	.26
Mental health	2.91	0.84	2.82	0.98	0.53	128.00	.60
Culture	3.35	0.48	3.28	0.52	0.81	126.71	.42
Resilience	4.20	0.54	3.99	0.78	1.76	129.00	.08
Help-seeking and helping	2.99	0.73	3.25	0.75	-1.98	121.59	.05
Peers	3.54	0.69	3.49	0.77	0.39	127.41	.70
Community	3.02	0.57	2.90	0.64	1.13	127.64	.26

Multiple *t*-tests were performed on the same data set. To avoid conflicts with family wise error rate, *t* scores and *p* values are presented without any claims of significance

## Results

### Sample

The entire sample consisted of only the youth (aged 11 to 18 at the time of Pre-Survey) who completed both a Pre- and a Post-Survey. There were 74 youth who completed both a Pre- and a Post-Survey in the IG. In the NIG, there were 59 youth who completed both a Pre- and Post-Survey. The average age of the IG (*M* = 13.5, *SD* = 1.5) did not differ significantly from the NIG (*M* = 13.3, *SD* = 1.4), *t*(129) = − 0.71, *p* = 0.482. These data, along with additional demographic information, are presented in Tables 2 and 3.

**Table 2:** Self-reported race and gender demographics

	NIG		IG	
	Percent	Count	Percent	Count
Race				
AIAN	98%	58	70%	52
Not AIAN	2%	1	30%	22
Gender				
Male	51%	30	51%	38
Female or non-binary	49%	29	49%	36

There was one non-binary person in the NIG and two in the IG; female and other category were grouped together (i.e., non-male) for analyses

**Table 3:** Age and risk score

	NIG		IG	
	Mean	*SD*	Mean	*SD*
ACE score	3.1	2.4	3.0	2.3
Age in years	13.3	1.4	13.5	1.5

It should be noted that 22 of the 74 (30%) youth in the IG did not self-identify as American Indian or Alaskan Native (AIAN) on the surveys (compared to only one in the NIG). When asked about this demographic information, one of the HOC curriculum facilitators noted that many Tribal families receiving Tribal services did not identify as Native despite their Native ancestry and Tribal member or descendant status. This pattern may be a result of intergenerational trauma due to colonization, which engendered internalized racism against Native Americans in some individuals. This history may be why many youth in the Native community choose not to identify as Native American. As such, this group of youth was retained in the sample due to ties to their Tribal communities.

There was modest attrition in the IG, with 74 youth completing the Post-Survey out of the 87 youth who completed the Pre-Survey (15% attrition). On the other hand, about half of the 129 youth in the NIG did not fill out a Post-Survey (54% attrition). This is likely because there was no intervention or programming to connect them with staff between Pre- and Post-Survey.

A repeated measures analysis of variance (rANOVA) was run for each of the following summary measures: hope, school, family, physical health, mental health, culture, resilience, and help-seeking and helping. A full presentation of the estimated marginal means from these models is presented in Table 4. These models included indicators for female and non-binary participants (i.e., non-male), indicators for those with an ACE score higher than 4, and age at the time of survey. ACE scores of 4 or more are associated with poorer mental health outcomes (Daníelsdóttir et al., 2024).

**Table 4:** Estimated marginal means from rANOVA for composite measure scores for IG versus NIG at Pre-Survey and Post-Survey

	NIG				IG			
	Pre		Post		Pre		Post	
	*EMM*	*SE*	*EMM*	*SE*	*EMM*	*SE*	*EMM*	*SE*
**Hope**								
Overall	4.35	0.18	4.22	0.21	4.11	0.13	4.40	0.15
Female or non-binary	4.38	0.20	4.10	0.24	4.14	0.16	4.28	0.18
**School**								
Overall	2.33	0.25	2.79	0.29	2.22	0.27	2.92	0.31
Female or non-binary	2.33	0.26	2.70	0.30	2.22	0.28	2.83	0.32
**Family**								
Overall	3.58	0.23	3.13	0.28	3.43	0.25	2.96	0.30
Female or non-binary	3.55	0.24	3.09	0.30	3.39	0.26	2.92	0.31
**Physical health**								
Overall	3.15	0.13	3.09	0.16	3.01	0.09	2.93	0.11
Female or non-binary	3.02	0.15	3.05	0.19	2.88	0.11	2.89	0.14
**Mental health**								
Overall	2.84	0.17	2.70	0.16	2.81	0.12	2.93	0.12
Female or non-binary	2.65	0.19	2.41	0.19	2.61	0.15	2.63	0.14
**Culture**								
Overall	3.29	0.09	3.38	0.10	3.28	0.07	3.36	0.08
Female or non-binary	3.28	0.10	3.36	0.12	3.27	0.08	3.34	0.09
**Resilience**								
Overall	4.20	0.12	3.92	0.13	4.00	0.09	4.06	0.10
Female or non-binary	4.17	0.14	3.83	0.16	3.97	0.11	3.97	0.12
**Help-seeking and helping**								
Overall	3.05	0.14	3.08	0.13	3.30	0.10	3.26	0.09
Female or non-binary	3.06	0.16	3.01	0.15	3.31	0.12	3.19	0.11
**Peers**								
Overall	3.60	0.38	3.52	0.41	3.50	0.40	3.51	0.43
Female or non-binary	3.48	0.39	3.38	0.42	3.39	0.42	3.38	0.45
**Community**								
Overall	2.88	0.11	3.00	0.12	2.83	0.08	3.07	0.09
Female or non-binary	2.98	0.13	3.01	0.14	2.93	0.10	3.07	0.11

The AIAN results for IG and NIG are not included here because there was only one individual that did not identify as AIAN in the NIG, and therefore these results are not helpful

Hope scale response options were (1) none of the time; (2) a little of the time; (3) some of the time; (4) a lot of the time; (5) most of the time; and (6) all of the time. School scale, family scale, peer scale, and community scale, and culture scale response options were (1) strongly disagree, (2) disagree, (3) agree, and (4) strongly agree. Physical scale and mental health scale response options were (1) poor; (2) fair; (3) good; (4) excellent. Resilience scale response options were (1) not at all, (2) a little, (3) somewhat, (4) quite a bit, and (5) a lot. Help-seeking and helping scale score response options were (1) very unlikely, (2) somewhat unlikely, (3) somewhat likely, and (4) very likely and (1) not confident at all, (2) not confident, (3) confident, and (4) very confident. Age was centered at 13 years of age for these estimated marginal means

An indicator comparing those who self-identified as AIAN from those who did not was also included, primarily for differentiating within the IG as there was little variation in AIAN within the NIG. While differences existed between the AIAN youth and non-AIAN youth in Pre- and Post-scores, they did not differ greatly from the overall means between Pre- and Post-. In other words, AIAN versus non-AIAN followed the same trends as the overall group for each individual composite score.

When these factors and covariates showed impactful differences, they are highlighted. Baseline Pre-Survey mean scores and the related t-tests across groups are outlined in Table 4. No notable differences exist between the Pre- scores for NIG and IG. A discussion of key results follows.

### Hope

**Fig. 1:**
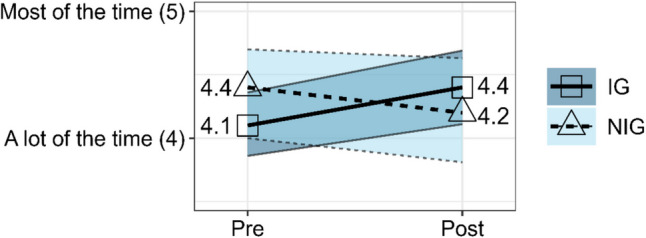
Estimated marginal means from rANOVA of composite hope scale score for IG versus NIG at Pre-Survey and Post-Survey with 95% confidence intervals. Figure graphics were created using the ggplot2 package in R (Wickham, 2016)

An rANOVA was conducted to compare the difference in the composite hope scale score between the IG and NIG from Pre-Survey to Post-Survey. The effect of group on the composite hope scale score was significant, *F*(1, 124) = 4.52, *p* = 0.036, partial *η*^2^ = 0.04, indicating a small to medium effect. As shown in Fig. 1, the estimated marginal means produced from the rANOVA show that the HOC intervention had a positive effect on the composite hope scale scores from Pre-Survey (*EMM* = 4.11, *SE* = 0.13) to Post-Survey (*EMM* = 4.40, SE = 0.15). The NIG saw a slight decrease in estimated marginal means from Pre-Survey (*EMM* = 4.35, *SE* = 0.18) to Post-Survey (*EMM* = 4.22, *SE* = 0.21).

### Mental Health

**Fig. 2:**
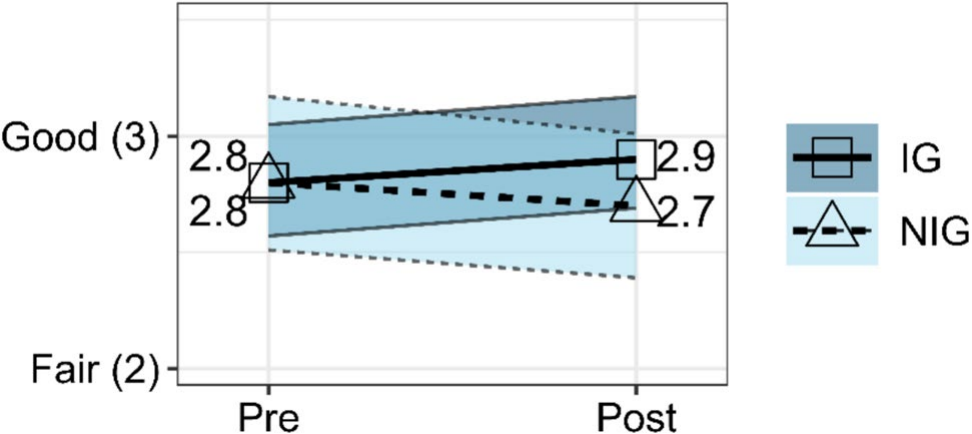
Estimated marginal means from rANOVA for mental health score for IG versus NIG at Pre-Survey and Post-Survey with 95% confidence intervals

The rANOVA for mental health between IG and NIG did not have a significant effect, *F*(1, 120) = 2.06, *p* = 0.154, partial *η*^2^ = 0.02. However, mental health scores from Pre-Survey (*EMM* = 2.81, *SE* = 0.12) to Post-Survey (*EMM* = 2.93, *SE* = 0.12) did increase slightly for IG participants, while mental health scores from Pre-Survey (*EMM* = 2.84, *SE* = 0.17) to Post-Survey (*EMM* = 2.70, *SE* = 0.16) decreased slightly for NIG participants as illustrated in Fig. 2.

**Fig. 3:**
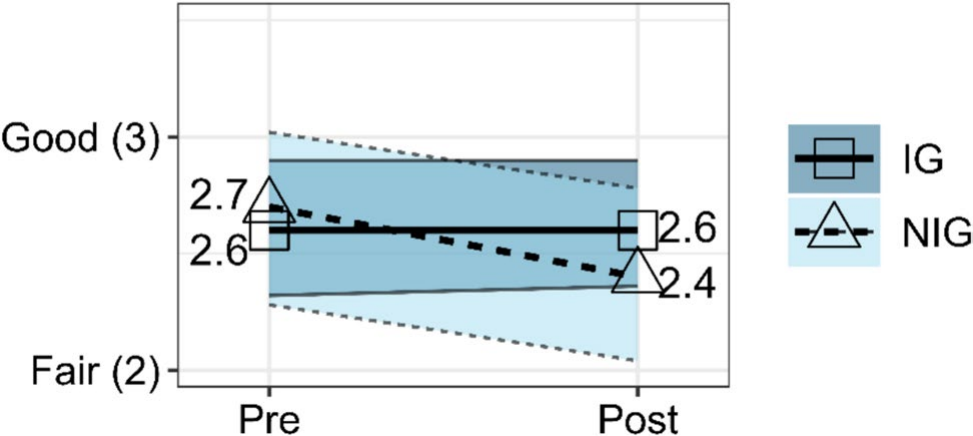
Estimated marginal means from rANOVA for mental health score for IG versus NIG, and youth identifying as female or non-binary, at Pre-Survey and Post-Survey with 95% confidence intervals

Regardless of intervention, female and non-binary participants (i.e., non-male) (*EMM* = 2.57, *SE* = 0.12) had lower mental health scores than male participants (*EMM* = 3.07, *SE* = 0.11) while controlling for age. However, while female and non-binary NIG participants saw decreases in mental health scores from Pre-Survey (*EMM* = 2.65, *SE* = 0.19) to Post-Survey (*EMM* = 2.41, *SE* = 0.19), IG female and non-binary participants remained stable from Pre-Survey (*EMM* = 2.61, *SE* = 0.15) to Post-Survey (*EMM* = 2.63, *SE* = 0.14) as shown in Fig. 3.

### Resilience

**Fig. 4:**
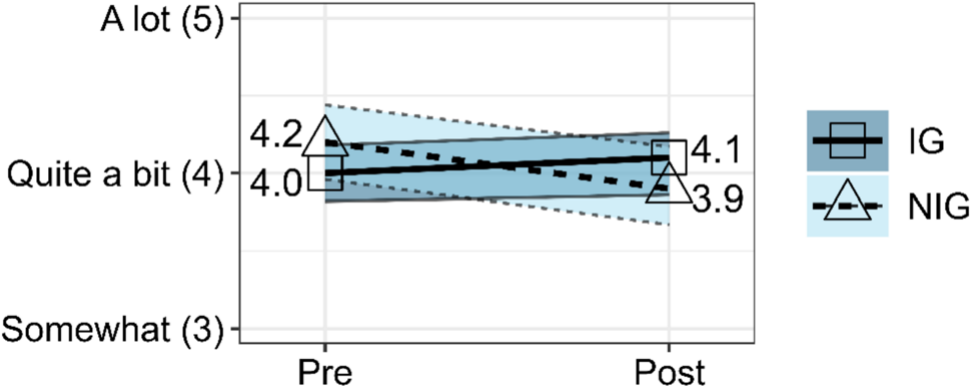
Estimated marginal means from rANOVA of composite resilience scale score for IG versus NIG at Pre-Survey and Post-Survey with 95% confidence intervals

The rANOVA for the composite resilience scale between IG and NIG had a significant effect, *F*(1, 122) = 7.42, *p* = 0.007, partial *η*^2^ = 0.06 indicating a large effect. Composite resilience scores from Pre-Survey (*EMM* = 4.00, *SE* = 0.09) to Post-Survey (*EMM* = 4.06, *SE* = 0.10) increased for IG participants, while composite resilience scores from Pre-Survey (*EMM* = 4.20, *SE* = 0.12) to Post-Survey (*EMM* = 3.92, *SE* = 0.13) decreased for NIG participants as illustrated in Fig. 4.

### Help-Seeking and Helping

**Fig. 5:**
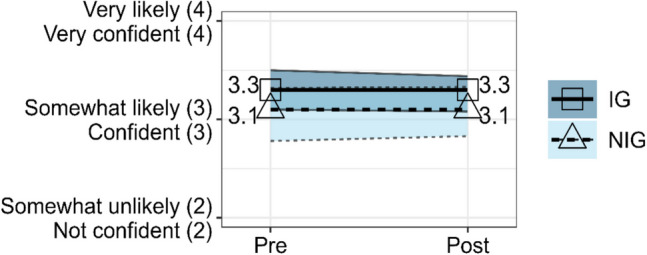
Estimated marginal means from rANOVA for composite help-seeking and helping scale score for IG versus NIG at Pre-Survey and Post-Survey with 95% confidence intervals. IG means went from 3.30 at Pre-Survey (Time 1) to 3.26 at Post-Survey (Time 2). NIG means went from 3.05 at Pre-Survey (Time 1) to 3.08 at Post-Survey (Time 2). Convergent rounding rules were applied in the plot labels

The rANOVA for the composite help-seeking and helping score between IG and NIG did not show a significant effect, *F*(1, 121) = 0.15, *p* = 0.702, partial *η*^2^ = 0.001. The composite help-seeking and helping score for the IG decreased negligibly from Pre-Survey (*EMM* = 3.30, *SE* = 0.10) to Post-Survey (*EMM* = 3.26, *SE* = 0.09). The NIG composite help-seeking and helping score similarly increased negligibly from Pre-Survey (*EMM* = 3.05, *SE* = 0.14) to Post-Survey (*EMM* = 3.08, *SE* = 0.13). In sum, the composite help-seeking and helping scores remained relatively stable from Pre- to Post-Survey for both groups, as shown in Fig. 5. The stability from Pre- to Post-Survey is also true when each of the three measures are observed alone rather than as a scale.

### Suicide attempts

Because the time lapse from Pre- to Post-Survey for the IG youth was less than 30 days, inferential analysis was not conducted (nor would rANOVA be the appropriate analysis).

With respect to the open-ended question about what the IG youth learned during the HOC curriculum, representative samples of youth responses from each of the four implementations included statements about how they learned patience, developed coping skills, gained cultural knowledge or skills, and made friends:

- “I learned patience, and I also have learned to grow into a better person”
- “Coping skills so I can let out my emotions”
- “I have a lot of people who love me”
- “How to carve wood”
- “It brought me closer to people and gave me new friends”
- “Every group has multiple stereotypes, and it is up to me to try and not get hurt by them”
- “I learned new things about my culture”
- “To take deep breaths”
- “Healthy relationships”

## Discussion

The focus of this study was exploring the effects of the HOC curriculum on hope, resilience, mental health, help-seeking, and helping. The findings of this study are consistent with the few other outcome studies of culturally-based suicide prevention and intervention programs that center on protective factors such as hope (Gray et al., 2019), resilience, (Hunter et al., 2022, which used the same CYMR- 12 measure as the present study), and self-esteem (Hunter et al., 2022; Kelley et al., 2018).

Many AIAN youth suicide prevention interventions focus on building connections to Native culture as a way to enhance protective factors against suicide. Researchers have found that cultural beliefs and experiences that lead to a life that is enjoyable, worthwhile, and provides meaning are protective factors against suicide (Allen et al., 2018). More recently, Allen and colleagues looked at “reasons for life” as an important protective factor against suicide in Alaskan *Yup’ik* youth (Allen et al., 2023). The current study included a culture scale, but it did not stand out in the IG/NIG comparison.

Youth who participated in culturally based Healing of the Canoe (HOC) classes experienced increased hope and resilience, as measured by self-report Pre- and Post-Surveys. Because this study was the first to evaluate the addition of the suicide prevention and intervention modules to the HOC curriculum, it is encouraging to see improvements in these two protective factors that are vital to mental wellness. There were some findings that, despite showing null results, may prompt future study, including slightly higher scores on the mental health scale for youth who participated in HOC than those who did not, and stabilization of mental health scores over time for female and non-binary youth who participated in HOC compared to decreases for female and non-binary youth who did not receive the curriculum. Because female and non-binary youth had lower mental health scores than male youth, it is especially beneficial to find ways to reverse downward trends in this key area.

Research in a community setting is both a strength and a challenge, and this study was no different. The strength is that the HOC program is an extension of the culture and values of the communities in which it was implemented, making HOC profoundly meaningful for Tribal leaders, HOC facilitators, and the participating youth. The challenge is that community-based interventions cannot be controlled as if they were in a laboratory.

As such, there are many important limitations to this study. There was no ability to dictate the location, timing, or format of the HOC curriculum delivery because each self-selecting Tribal site chose the location, timing, and format that would work best for their staff and youth participants (i.e., spring break camp versus quarter-long class at school). Thus, the timing of the HOC Pre- and Post-Surveys also varied according to the timing/format of HOC delivery. Nor was the study team able to control the timing of the NIG Pre- and Post-Surveys because these data were not originally envisioned to be part of a larger comparison study. This meant that the time-lapse within the HOC sample Pre- and Post-Surveys and between the NIG was substantially varied. Another limitation is that measures were self-reported by youth, and many comparison group youth were lost to follow-up. Additionally, there were many youth in the HOC group who did not self-identify as AIAN. HOC staff noted that these youth were from the Tribal community and that many Tribally-enrolled adults themselves do not identify as AIAN. It is sadly conceivable to imagine that this is the result of internalized negative views of Native people handed down historically and still in place today. Without additional information, of course, this explanation is unsubstantiated.

Despite the limitations, programs like HOC that teach coping skills and incorporate cultural elements show promise in positively impacting youth mental health. HOC helps counter poor mental health trends among youth and risk factors resulting from difficult life experiences. Youth who did not participate in HOC experienced decreases in hope and resilience over time, and the HOC intervention appears to have interrupted that trend and moved youth in a positive direction.

Future studies that increase the number of youth in both intervention and comparison groups would be beneficial to see if the encouraging patterns reach significance with greater statistical power. Gathering information about the dosage of the intervention each youth received, and more details about how the curriculum was delivered, would also be immensely useful. Tribal communities implement HOC in diverse ways, such as in after-school programs, during the school day, on weekends, and during school breaks. Determining whether there are delivery strategies that are more effective than others or the extent to which involvement in complementary cultural activities enhances the benefits of the curriculum could improve HOC for future cohorts.

It would be valuable to explore the areas in this study that did not see change (such as culture, help-seeking, and helping measures) and whether there are enhancements that could be made to the curriculum through additional staff training, content, exercises, or modules, to address youth needs and build on strengths and skills. Additional data collection could assess if, for instance, youth in the IG were already exposed to suicide prevention education or if different but related material, such as building confidence and practicing asking for help, would lead to changes in these areas.

Despite a small number of youth participants and the naturalistic setting that limited the ability to control for potential confounding variables, the results from this preliminary study are promising. HOC appears to have a positive impact on youth in key areas. It would be worthwhile to invest in continuing to offer this curriculum to additional youth and communities as well as to gain knowledge of how to maximize its effectiveness. In sum, this preliminary study documented positive HOC impact in diverse PNW community settings over varied implementation types, which is an important step to help substantiate the need for more rigorous—and also culturally sensitive, ethical, and responsive—studies.

## Data Availability

The dataset and code may be available upon request. Please contact Colbie Caughlan at ccaughlan@npaihb.org. Requestors must disclose what they plan to do with the data. Release will be subject to Portland Area Indian Health Service IRB approval. Requestors must pledge not to further disclose or misuse the data.
